# Design and Implementation of C-Band Large-Power Planar Butler Matrix in SRS

**DOI:** 10.3390/s24072132

**Published:** 2024-03-27

**Authors:** Jinfeng Li, Liping Yan, Changjun Liu, He Bai, Wanzhao Cui

**Affiliations:** 1 The School of Electronics and Information, Sichuan University, Chengdu 610064, China; 2 National Key Laboratory of Science and Technology on Space Microwave, China Academy of Space Technology, Xi’an 710049, China

**Keywords:** Butler matrix, multipactor, power threshold, short-ended stubs, square coaxial line

## Abstract

In satellite remote sensing (SRS), there is a demand for large-power microwave components. A Butler matrix is essential to a transmitting antenna array in SRS. This article illustrates the electrical and mechanical design, simulation, and test results of a large-power planar beamforming network for SRS at C-band. It is a 4 × 4 Butler matrix based on square coaxial lines. Short-ended stubs are used in the Butler matrix to broaden its bandwidth by 10%, support inner conductors, and enhance heat transfer in vacuum environments. The simulation results are consistent with the measured results. The reflection coefficient is less than −18 dB, and the isolation is more than 23 dB from 3.8 GHz to 4.2 GHz. The insertion losses are less than 0.6 dB, and the phase errors are better than ±6°. The measured peak microwave power of the proposed Butler matrix is 9 kW. Its size is 440 × 400 × 40 mm^3^. The proposed Butler matrix beamforming network can be applied to SRS systems.

## 1. Introduction

SRS needs to detect ground objects in space and is characterized by long distance and multiple targets, which requires large transmitting microwave power and multi-beam-transmitting antennas. Therefore, studying the method of realizing multi-beam antenna array and exploring a large-power transmission line structure is necessary.

The beamforming networks are an essential part of an SRS transmitting antenna array. There are many beamforming networks reported [1], like the Rotman lens, Blass matrix, Nolan matrix [2], and Butler matrix [3,4,5]. Compared with these passive beamforming networks, the Butler matrix is the most popular choice, given its more straightforward and cost-effective structure and wider bandwidth [6]. In addition, the Butler matrix has the advantages of reliability and reciprocity; therefore, it is widely used in several applications, such as SRS, Internet of Things (IoT), Wi-Fi, base stations, satellite communications, and automotive radars [3].

The study of the Butler matrix [7,8,9] involves many aspects, such as the perspective of the frequency band, 77 GHz [3], Ka-band [10], K-band [11], X-band [12], C-band [13], and S-band [13]. From the perspective of large-power design [14], a specific computer-aided method for a large-power Butler matrix design is given [15]. Various realization techniques and associated transmission line technologies have been reported to fabricate Butler matrices. These include microstrip lines [16], waveguides [14], and square coaxial lines [17], to list a few examples. Substrate-integrated waveguide (SIW) Butler matrix has been developed as a compromise between the low loss and good high-frequency performances of rectangular waveguide types, as well as the integrability of planar transmission line types. However, the most challenging problem of the Butler matrix design is the presence of path crossings, which may lead to large dimensions when directly using SIW [7]. The fabrication of the substrate-integrated waveguides is complex and expensive [3]. In some contexts, it is preferred to microstrip technology for its higher immunity to interference, no air radiation, and lower dispersion. However, realizing coaxial RF components with variable line impedances is generally inconvenient with traditional manufacturing technologies [9]. The multipactor mechanism of introducing dielectrics between metal plates as dielectric-filled waveguides is analyzed. However, breakdown and thermal heating are potential challenges of using dielectric materials in a high-power radio frequency (RF) environment [18]. There is no size advantage at all over highly integrated transmission lines such as microstrip lines and substrate-integrated waveguides, but coaxials and waveguides sacrifice size to achieve kW levels of power, while other transmission lines reach only mW to W levels of power.

In [12], a miniaturized large-power beamforming network using an 8 × 8 Butler matrix based on waveguides was successfully designed and tested for an X-band synthetic aperture (SAR) system. The insertion loss and isolation of the unit are better than 0.4 dB and 25 dB, respectively, over 600 MHz bandwidth at 9.6 GHz. The measured average and peak microwave power are 500 W and 6 kW, respectively. The whole unit was machined with a modified non-planar structure with a mechanical size of 478 × 362 × 264 mm^3^.

In [16], a PCB-based beamforming network was designed for Ka-band applications. Due to the low power capacity of the PCB structure, the measured power value was only 0.01 kW. Finally, by combining multiple phase shifters through a new design approach, a millimeter-wave beamforming network is introduced to achieve beam-steering antenna arrays. The beam steering is performed with a single microfluidic actuator and a circuit model is introduced to facilitate the design of the beamforming network. The design example presented is for a four-element antenna array operating at 28 GHz, exhibiting continuous beam steering capability within ±30° when its SMP is actuated within its −100 to 100 μm displacement range. Unlike beam-steering antennas using beamforming ICs, they do not require active amplification to compensate for high loss and have a reduced hardware complexity associated with control bits and bias lines. Compared to previously investigated microfluids-actuated beam-steering antennas, it offers the advantage of not relying on switched beam techniques and bulky lenses.

In [17], a square coaxial line beam former was designed. Simulation results demonstrated that the square coaxial line could reach a power capacity of 8 kW in C-band. Impedance matching was achieved by creating a groove discontinuity in the inner conductor. A cone was provided for smooth impedance transformation for required broad banding. The measured performance of the beam former was obtained by feeding it to the feed layer of the electromagnetically coupled multilayer patch antenna. Equal and unequal power dividers were designed with a proper impedance transformer by changing dimensions of the inner conductor but keeping same outer conductor dimensions. Smooth linear taper was provided at the impedance transformer junction. High power calculations were also presented for square coaxial lines to handle several kW of power.

In this work, a large-power planar Butler matrix is implemented in C-band. Compared to prior works, this work advances the wide bandwidth design and square coaxial line for easy machining. In this paper, the aim of designing a 4 × 4 square coaxial Butler matrix is to divide the overall structure, using four steps to complete it. Firstly, the key component, the orthogonal coupler, is designed. The design is mainly concerned with the isolation, i.e., the coupling between input ports, to ensure that the Butler matrix performance from deterioration. Then, the cross junction is optimized with the design of the coupler cascade, followed by the design of the phase shifter and the cross junction. With tunning of the cross junction, the output phase balance is optimized, which also ultimately determines the phase stability of the Butler matrix. Finally, according to the schematic diagram, performance indexes, such as isolation, output amplitude flatness, phase flatness, and others, are optimize with the comprehensive consideration of the inhibition of the multipactor. It finally results in a well-working Butler matrix.

## 2. Principle and Design Method

### 2.1. Principle

**Figure 1:**
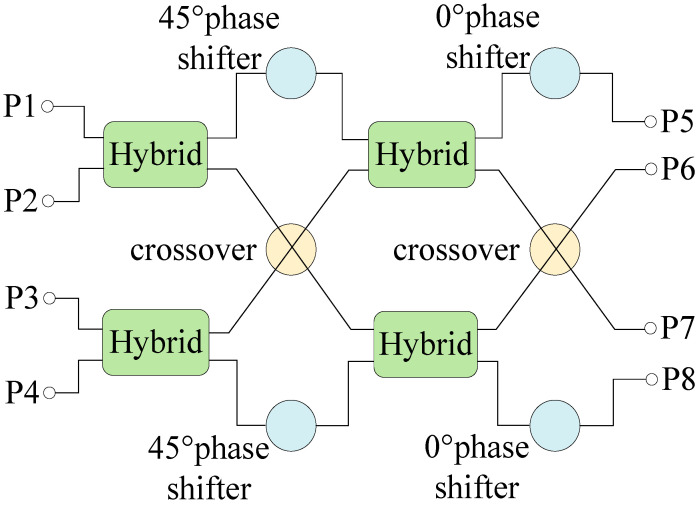
Schematic of a 4 × 4 Butler matrix.

A Butler matrix is a beamforming network [19]. Figure 1 shows the schematic of a 4 × 4 Butler matrix designed in this article. The input signal at input ports P1 to P4 is redistributed in the Butler matrix through four 3 dB hybrid couplers, two crossovers, two 45° phase shifters, and two 0° phase shifters. The Butler matrix delivers signals to its output ports with equal magnitudes and a phase increment between them of 45°, −135°, 135°, and −45° depending on which input port is excited. The phase difference between output ports is fixed when any input port is fed. The wideband performance of a Butler matrix is mainly due to the wideband design of its components, e.g., couplers, crossovers, and phase shifters.

### 2.2. Square Coaxial Line

#### 2.2.1. Design of Square Coaxial Line

Butler matrices are designed using various transmission lines, such as waveguides, microstrip lines, and coaxial lines [20]. When the operating band is in the C-band, the rectangular waveguide size of BJ40 is significantly larger than the square coaxial structure of our design. The coaxial line has the advantage that the signal is shielded by the outer conductor. Therefore, the coupling to the adjacent component can be avoided. A suppression multipactor concept is considered to use the TEM mode in the coaxial line. A square coaxial line is easier to machine than a cylinder coaxial line in a Butler matrix.

**Figure 2:**
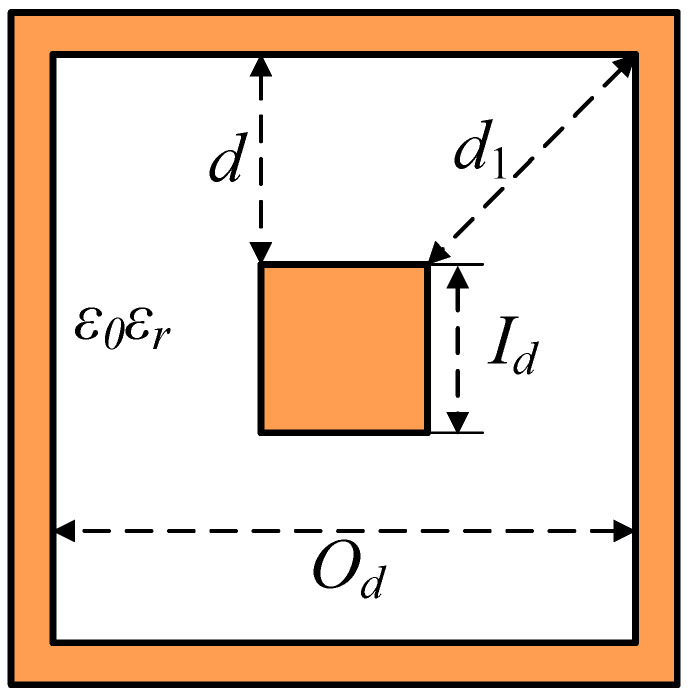
Square coaxial line cross-section structure.

Two conditions are met in the design: (1) characteristic impedance should be 50 Ω to match the input and output terminals of the system; and (2) low attenuation. The design of a beam former begins with evaluating the inner and outer diameter to achieve the distance between them for handling higher microwave power levels. The characteristic impedance depends on the ratio of outer and inner conductor diameters. For a 50 Ω square coaxial, the inner conductor used in the square coaxial line is 4.8 mm, and the outer conductor is 12 mm. The cross-section of a square coaxial line is shown in Figure 2. The white is the vacuum, and the orange is aluminum.

Its characteristic impedance [21] can be approximately calculated by

(1) $$Z_{0}=\frac{136.7}{\sqrt{\varepsilon_{r}}}\mathrm{lg}\left(0.9259\frac{O_{d}}{I_{d}}\right)$$

Its attenuation constant [21] can be approximately calculated by

(2) $$\alpha_{c}=\frac{59.37R_{s}}{\eta_{0}Z_{0}}\left(1+\frac{I_{d}}{O_{d}}\right)\frac{1}{O_{d}}$$

where *O*_d_ and *I*_d_ are the outer and inner conductor diameters, respectively, and *ε*_r_ is the relative dielectric constant of the vacuum. *O*_d_ is 12 mm, and *I*_d_ is 4.8 mm, as presented in Figure 2, which leads to *O*_d_/*I*_d_ is 2.5.

#### 2.2.2. Electrical Performance Simulation

**Figure 3:**
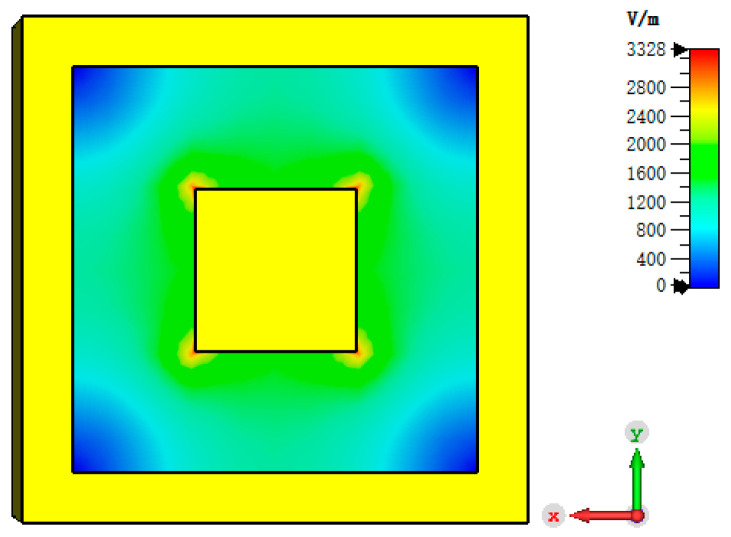
E-field distribution of square coaxial line.

**Figure 4:**
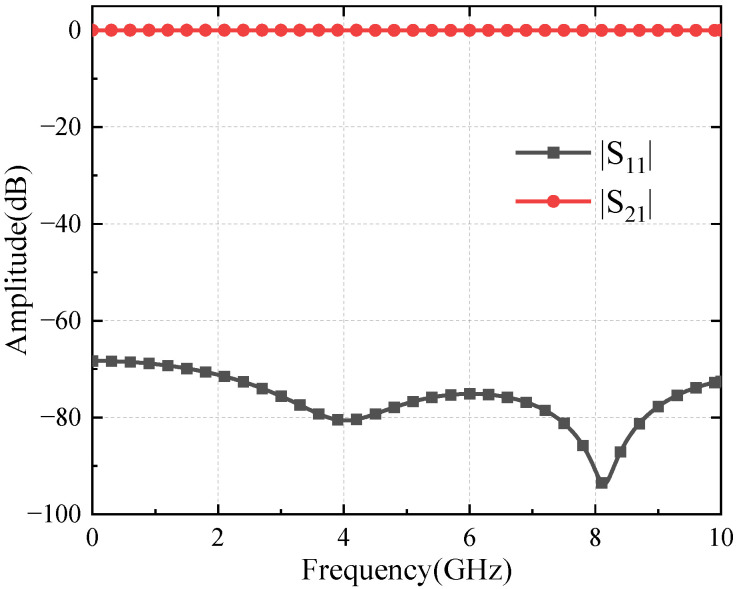
Simulation results of square coaxial line S-parameter.

Use the 3D electromagnetic simulation software CST Studio Suite 2020 to simulate the square coaxial line. The length is 1000 mm, and the material is aluminum. The electric field distribution is entirely symmetrical. The highest electric field intensity is located on the edge of the inner conductor, and the peak electric field is about 3500 V/m, as shown in Figure 3. The insertion loss is less than 0.01 dB within a given frequency range, and the return loss is greater than 65 dB, as shown in Figure 4.

#### 2.2.3. Multipactor Simulation

**Figure 5:**
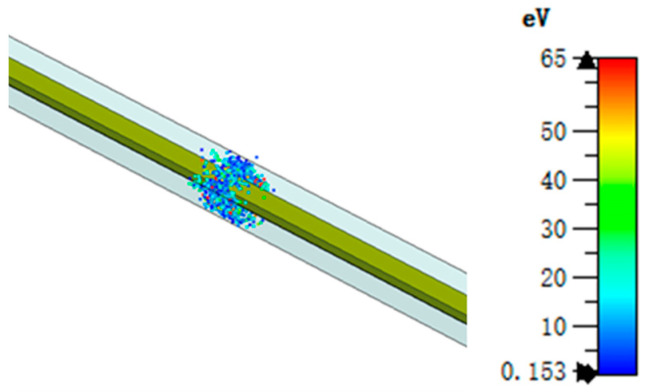
Simulation model of square coaxial line multipactor.

**Figure 6:**
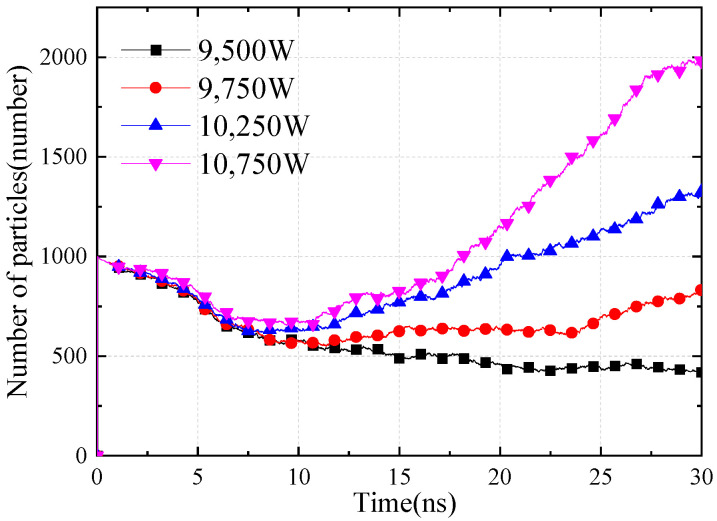
Simulation results of square coaxial line multipactor threshold.

Figure 5 shows the square coaxial line’s multipactor [22] simulation model. The initial particle cloud is located in the middle of the square coaxial line, the initial number of particles is 1000, the simulation time is 30 ns, and the center frequency is 4 GHz. The square coaxial line multipactor threshold simulation result is about 9500 W in Figure 6.

When other conditions are guaranteed not to change, the frequency offset will also change the multipactor threshold power. The threshold power is related to *f* × *d*, where *f* is the frequency and *d* is the distance between two metals.

### 2.3. Design and Analysis

#### 2.3.1. Coupler Analysis

The most central component of the Butler matrix is the quadrature coupler, also known as the branchline hybrid network. The loaded short-ended stubs coupler was designed using square coaxial lines. The branch coupler’s scattering parameters are

(3) $$\left[S\right]=-\frac{1}{\sqrt{2}}\left[\begin{array}{cccc} 0 & j & 1 & 0\\ j & 0 & 0 & 1\\ 1 & 0 & 0 & j\\ 0 & 1 & j & 0 \end{array}\right]$$

Its scattering matrix has symmetry, and the values at the diagonal are all zero to characterize that all four ports are matched. The design of the orthogonal coupler focuses on the following parameters: input reflection coefficient, isolation, transmission coefficients, output ports phase difference, bandwidth.

When cascaded in the Butler matrix, the branch coupler is not sufficiently flat in its in-band output amplitude to narrow the available bandwidth. Short-ended stubs are loaded at the four ports of the branch coupler to extend the bandwidth of the branch coupler and improve isolation.

**Figure 7:**
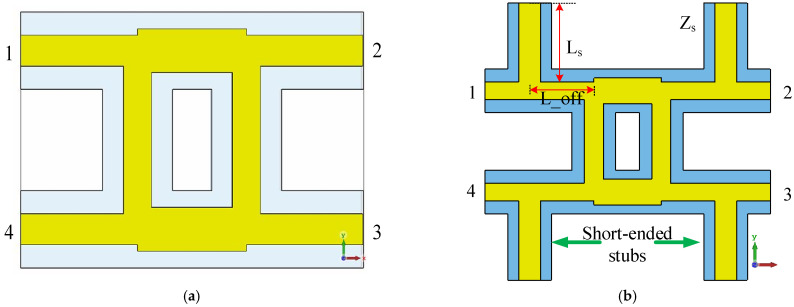
Simulation model of the coupler (**a**) without short-ended stubs; (**b**) with short-ended stubs.

**Figure 8:**
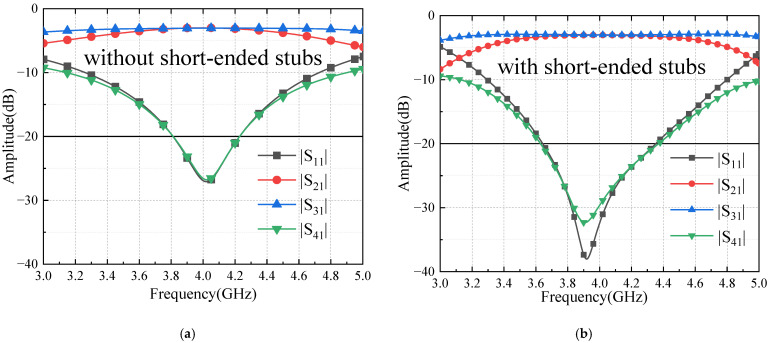
Simulation results of coupler S-parameters (**a**) without short-ended stubs; (**b**) with short-ended stubs.

Short-ended stubs [23] are used in Butler matrix components to broaden the bandwidth by 10%. Meanwhile, they help heat dissipation and prevent device burnout. Four pairs of short-ended stubs are placed to support the inner conductors, broadening the bandwidth [24,25,26] and not affecting the output magnitude. The couplers are optimized for impedance matching. The simulation model is shown in Figure 7. Ls = 21.7 mm, L_off = 19 mm. The simulation results are shown in Figure 8.

#### 2.3.2. Crossover Analysis

The crossover is an important connecting unit in the Butler matrix. It is also a symmetrical four-port device and operates in such a state that when one of the ports is input, the diagonally opposite output port is at equal amplitude, and the remaining two ports are isolated. The crossover scattering parameters are

(4) $$\left[S\right]=\left[\begin{array}{cccc} 0 & 1 & 0 & 1\\ 1 & 0 & 1 & 0\\ 0 & 1 & 0 & 1\\ 1 & 0 & 1 & 0 \end{array}\right]$$

**Figure 9:**
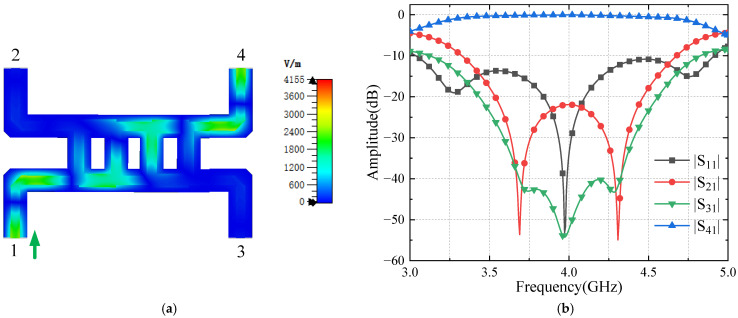
Simulation results of the crossover (**a**) E-field distribution; (**b**) S-parameters.

Two unloaded short-ended-stub hybrid couplers can be cascaded to create a Butler matrix crossover. Direct synthesis using a broadband coupler would significantly increase the overall size of the Butler matrix. The signal transmission path shows that when input port 1 is fed, output port 4 has equal amplitude output, and both output port 2 and input port 3 are isolated states modeled in Figure 9a. The scattering parameter simulation results are shown in Figure 9b; it can be seen that the isolation is more than 20 dB in the frequency range of 3.5–4.5 GHz, the insertion loss is less than 0.05 dB, and the curve shows symmetry centered at 4.0 GHz. The input VSWR is less than 1.2 in the frequency range of 3.8–4.2 GHz.

#### 2.3.3. Phase Shifter Analysis

**Figure 10:**
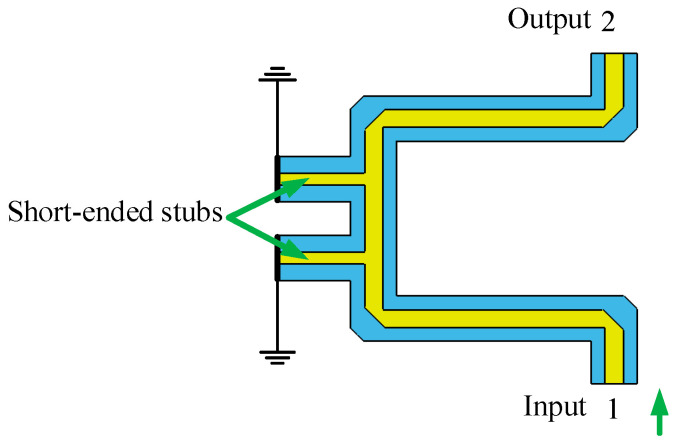
Simulation model of 45° phase shifter.

**Figure 11:**
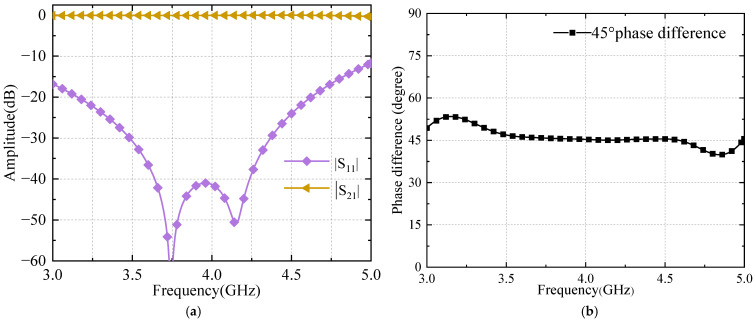
Simulation results of 45° phase shifter (**a**) S-parameters; (**b**) phase difference.

**Figure 12:**
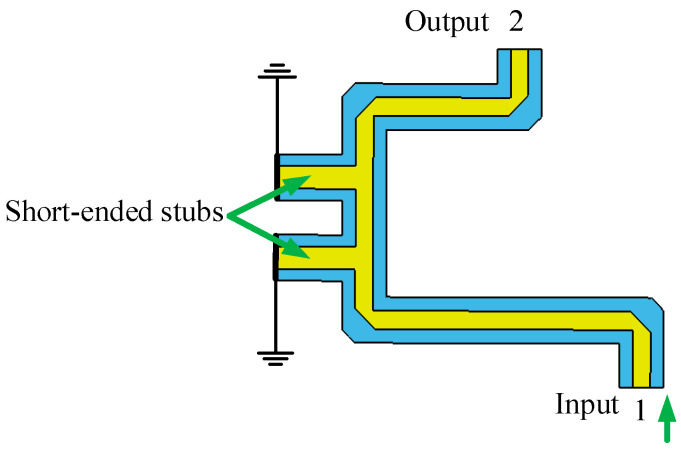
Simulation model of 0° phase shifter.

**Figure 13:**
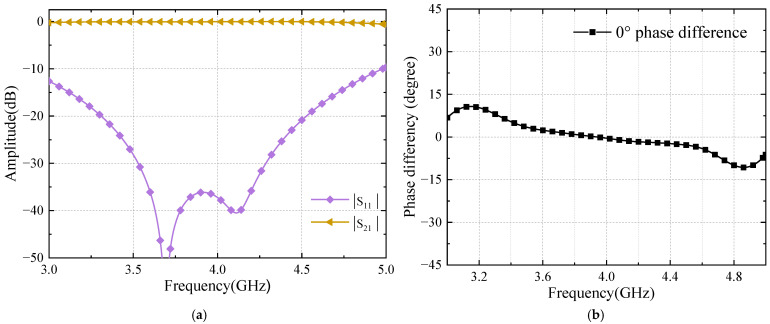
Simulation results of 0° phase shifter (**a**) S-parameters; (**b**) phase difference.

A phase shifter [27] with good phase flatness in the operational frequency band directly determines the accuracy of the Butler matrix output phases. The lengths of the phase shifters and the interconnecting lines have been tuned to offset the phase change caused by the stubs. The broadband 45° phase shifters and 0° phase shifters have been realized. The simulation model of phase shifters and simulation results are shown in Figure 10, Figure 11, Figure 12 and Figure 13, respectively.

The wideband 45° phase shifter has good transmission performance at a frequency of 3.6–4.2 GHz with a return loss greater than 40 dB and an in-band insertion loss less than 0.1 dB. The output phase difference between them is 45 ± 1°, and the phase flatness is superior. The simulation results are shown in Figure 11.

The wideband 0° phase shifter has good transmission performance at a frequency of 3.6–4.2 GHz with a return loss greater than 30 dB and an in-band insertion loss less than 0.14 dB. The output phase difference is 0 ± 1.5°. The simulation results are shown in Figure 13.

#### 2.3.4. Butler Matrix Design

The Butler matrix was designed as an eight-port device. The input ports are labeled P1 to P4, and the output P5 to P8. The rest of the details of the design are chamfered edges, ventilation holes, square coaxial line corners for corner-cutting, square coaxial line corners for rounding, avoiding low-pressure discharge, etc. These design factors were considered to achieve high power threshold.

**Figure 14:**
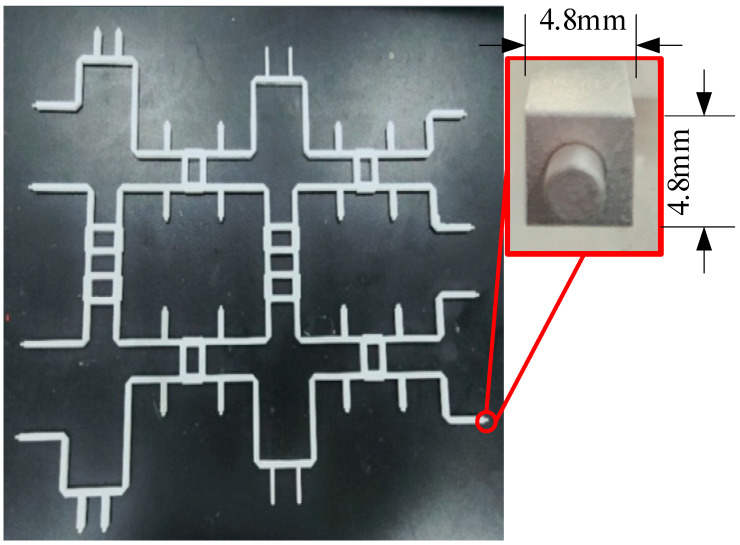
The fabricated Butler matrix’s inner conductor.

**Figure 15:**
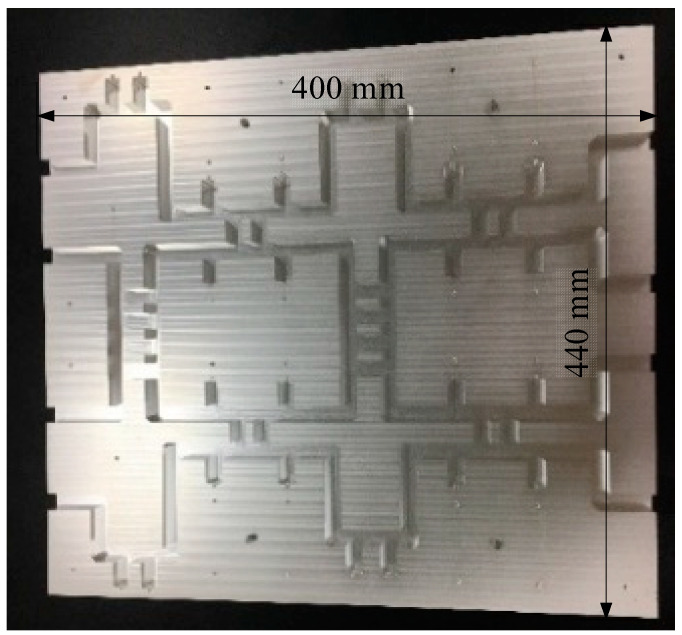
The fabricated Butler matrix’s outer conductor.

Aluminum alloy is used to build both inner and outer conductors. The material used in the simulation of Butler matrix is aluminum, and the actual machining is performed with aluminum alloy in order to machine the material more rigidly. One significant detail is that each of the eight ports of the Butler matrix leads to a small cylinder in order to facilitate testing with the L29 connector. The cylinder size is 2 mm in diameter and 4 mm in height. The proposed Butler matrix [28] uses a planar open-cover structure, as shown in Figure 14 and Figure 15. The mounting configuration ensures the strength of the structure. It is 440 mm by 400 mm and has a height of 40 mm.

## 3. Results

### 3.1. Simulation

The optimally designed coupler, crossover junction, 45° phase shifter, and 0° phase shifter are applied to the Butler matrix beamforming network, and the entire model is electromagnetically simulated to analyze the performance, e.g., port reflection coefficients, input and output port isolations, transmission coefficients, and output port phase differences. Due to the model’s symmetry, the scattering parameters related to input port 1 and input port 4 are identical when the input port is fed. Similarly, this works for input port 2 and input port 3 as well. The phase difference between the output ports is 45°, −135°, 135°, and −45°, respectively.

**Figure 16:**
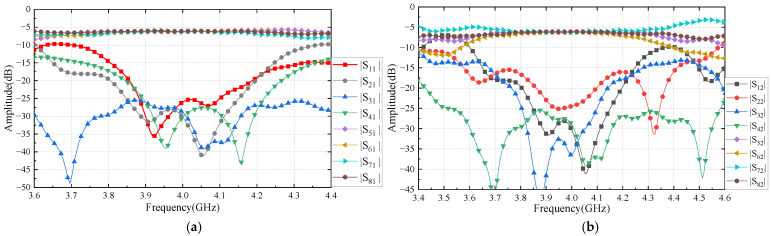
Simulation results of Butler matrix input ports’ scattering parameters: (**a**) reflection coefficient of input port 1 and transmission coefficients of input port 1 to other ports; (**b**) reflection coefficient of input port 2 and transmission coefficients of input port 2 to other ports.

When input port 1 and input port 2 are fed separately, the input reflection coefficient, transmission coefficient, and isolation of the Butler matrix beamforming network are shown in Figure 16. In the frequency range of 3.85–4.15 GHz, the input reflection coefficient of input port 1 is less than −20 dB, the input isolation is greater than 20 dB, and the transmission coefficient is −6.0 dB ± 0.4 dB. The input reflection coefficient of input port 2 is less than −20 dB. The isolation between input ports is greater than 20 dB, and the transmission coefficient is −6.0 dB ± 0.4 dB. The insertion loss of the matrix is small. It shows that the performance of the Bulter matrix is close to an ideal matrix.

**Figure 17:**
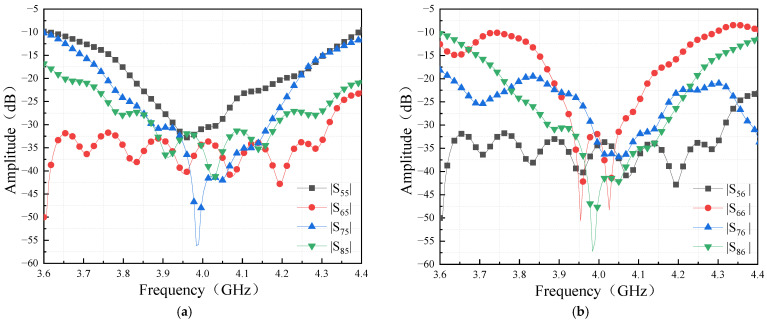
Simulation results of Butler matrix output ports scattering parameters: (**a**) reflection coefficient of output port 5 and isolation of output port 5 to other ports; (**b**) reflection coefficient of output port 6 and isolation of output port 6 to other ports.

The isolations between the output ports are greater than 20 dB in the frequency range of 3.8–4.2 GHz, as shown in Figure 17. The high isolation between output ports guarantees the independence of each output port, which will benefit the phased array for beamforming. Meanwhile, the reflection coefficients of output port 5 and output port 6 are less than −20 dB as well. The Bulter matrix’s output port is matched to 50 Ω, i.e., standard antennas connected to the output port will avoid impedance mismatch and prevent large microwave power being reflected to the Butler matrix.

**Figure 18:**
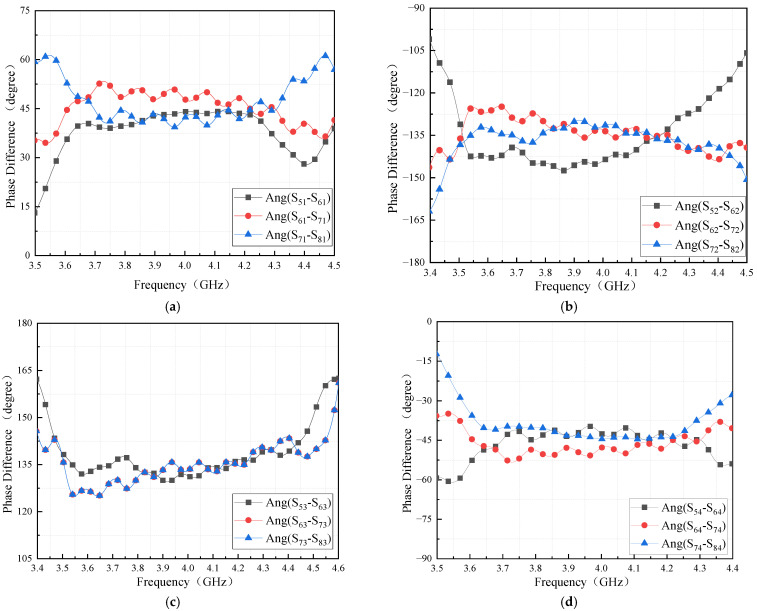
Simulation results of Butler matrix phase difference: (**a**) phase difference of input port 1; (**b**) phase difference of input port 2; (**c**) phase difference of input port 3; (**d**) phase difference of input port 4.

When the output port is fed, the phase difference between the output ports is in the frequency range of 3.9–4.1 GHz, as shown in Figure 18, and the phase difference between neighboring output ports is 45 ± 5° when input port 1 is fed. The phase difference between neighboring output ports is −135 ± 5° when input port 2 is fed. The stabilized phase frequency range is shifted to the lower frequency when input port 3 is fed, and the phase difference between neighboring output ports is 135 ± 10°. The phase difference between neighboring output ports is −45 ± 6° when input port 4 is fed.

### 3.2. Measurement

**Figure 19:**
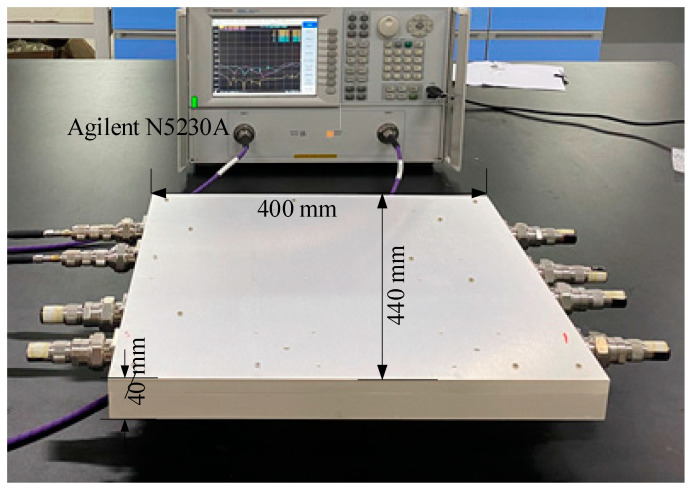
The Butler matrix measurement system.

A vector network analyzer (VNA, Agilent N5230A, Agilent, Santa Clara, CA, USA) was applied to measure the S-parameter. Figure 19 shows the Butler matrix S-Parameters measurement system. For the first measurement, two ports of the Butler matrix are connected to two cables of the vector network analyzer, and the rest of the ports are connected to N-matched loads.

**Figure 20:**
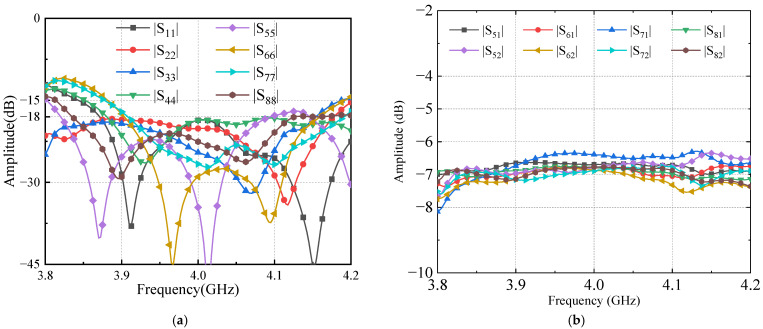
Measured results of Butler matrix S-parameters: (**a**) ports’ reflection coefficients; (**b**) input ports’ transmission coefficient.

**Figure 21:**
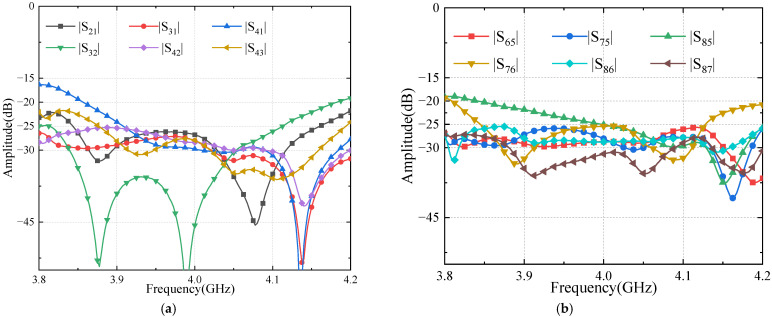
Port isolation of the Butler matrix (**a**) input ports; (**b**) output ports.

**Figure 22:**
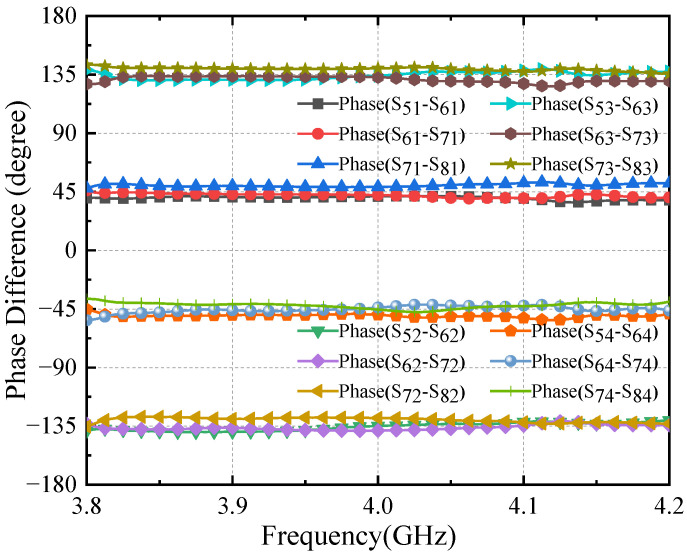
The phase difference of the Butler matrix.

The test results are shown in Figure 20, Figure 21 and Figure 22, respectively, and are consistent with the simulation results. Figure 20a shows that the reflection coefficients of ports are better than −18 dB. Figure 20b presents measured transmission coefficients when the Butler matrix is fed at input port 1 and input port 2. The coupling coeffects are well equalized, around −7 dB between 3.8 and 4.2 GHz.

Figure 21a,b show the measured results of the isolations between the input and output ports. In the band of 3.9–4.1 GHz, the isolations are close to our goal of 25 dB. Moreover, the measured results agree with the simulations well.

Figure 22 shows the measured phase differences for the four input ports. The phase differences between different output ports when the signal is fed at each input port are 45° ± 6°, −135° ± 5°, 135° ± 6°and −45° ± 6° over the frequency range. Good agreement between simulations and measurements is found, although the measurement results are slightly worse than the simulation. The machining error of the short-ended stubs impacts the phase shift and will also stress the test connector. The machining parameter error of 0.4 mm is out of the range of machining accuracy and causes a phase error of about 2°.

**Figure 23:**
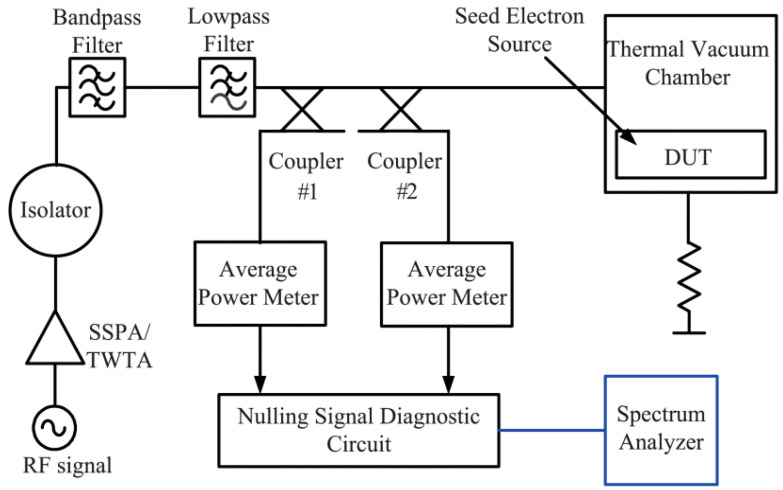
The experimental test setup of multipactor.

Figure 23 shows the experimental [29,30,31] setup of the multipactor measurement [18], which includes the data acquisition and processing system. A portion of the incident power applied to the device under test is extracted, fed into a coupling circuit, and nulled against a part of the extracted reflected power. Power detection is performed using average power meters (Agilent E14419B), and the data recording rate is 1000 times per second. A traveling wave tube amplifier (TWTA) is applied to provide large microwave power. We adopt nulling of the forward/reverse power detection method to conduct multipactor experiments in test samples. The test frequency is 4 GHz. Using a dual-directional coupler, the incident and reflected microwave powers are sampled.

After realizing power-level zeroing, the spectrum analyzer monitors the zeroed signal [32] in real time. The multipactor threshold can be tested by pulse modulation signal with a duty cycle of 10%. Thirty minutes should be allotted for each test power level till breakdown occurs. Nulling signal level of forward/reverse power is less than −60 dBm when test samples work correctly. Nulling signal level will increase as soon as the multipactor takes place.

In addition, these test samples must be cleaned with alcohol before they are mounted in a vacuum. The temperature of the test sample must be monitored during the experiment. The air pressure in the vacuum must be less than 1 × 10^−6^ Torr and remain for more than 24 h until the experiment begins. Radioactive source Cesium 137 was used to produce sufficient electrons. The multipactor microwave power threshold was tested to be 9 kW.

The bandwidth of the Butler matrix is significantly better than that of other research works listed in Table 1, reaching 10%. According to the product of frequency and distance, the measurement results of multipactor microwave power threshold based on the square coaxial line Butler matrix is also good.

## 4. Conclusions

To solve the problems of large-power transmission and planar structure, it is proposed to design a Butler matrix beamforming network based on a square coaxial line. Square coaxial lines are easily fabricated and have a high microwave power threshold compared with circular ones. Shunt short-ended stubs on the branchline coupler are adopted in the strategy, which can increase the bandwidth of a conventional one by 10%. The whole Butler matrix with introduced short-ended stubs is simulated and optimized. The matrix with a 400 mm by 400 mm dimension was fabricated and measured. The measured results are consistent with those of the simulations. The measurement results of the developed large-power 4 × 4 Butler matrix 9 kW are presented.

In follow-up work, further research will be conducted on a compact design and widening the microwave transmission bandwidth. Moreover, we will coat the inner conductors with special materials to decrease the electron emission coefficient, and thus further enhance the microwave power threshold of the matrix. It could be used for large-power Butler matrices for SRS applications.

## Figures and Tables

**Table 1:** Performance with the prior Butler matrix.

Refs	f_0_/GHz	d_min_/mm	20 dB-Bandwidth	Configuration	Power/kW
[16]	28	0.203	5.7%	1 × 4 PCB	0.01
[12]	9.6	10.16	6.25%	8 × 8 WR90	6
[17]	5	2.75	4%	1 × 8 square coaxial line	8
This Work	4	2.35	10%	4 × 4 square coaxial line	9

## Data Availability

Data are contained within the article.
